# Mechanistic insights into the interactions between cancer drivers and the tumour immune microenvironment

**DOI:** 10.1186/s13073-023-01197-0

**Published:** 2023-06-05

**Authors:** Hrvoje Misetic, Mohamed Reda Keddar, Jean-Pierre Jeannon, Francesca D. Ciccarelli

**Affiliations:** 1 Cancer Systems Biology Laboratory, The Francis Crick Institute, London, NW1 1AT UK; 2 School of Cancer and Pharmaceutical Sciences, King’s College London, London, SE11UL UK; 3 Department of Head & Neck Surgery, Great Maze Pond, Guy’s Hospital, London, SE1 9RT UK

**Keywords:** Cancer driver genes, Cancer immunology, Computational biology, Head and neck cancer, Functional networks

## Abstract

**Background:**

The crosstalk between cancer and the tumour immune microenvironment (TIME) has attracted significant interest in the latest years because of its impact on cancer evolution and response to treatment. Despite this, cancer-specific tumour-TIME interactions and their mechanistic insights are still poorly understood.

**Methods:**

Here, we compute the significant interactions occurring between cancer-specific genetic drivers and five anti- and pro-tumour TIME features in 32 cancer types using Lasso regularised ordinal regression. Focusing on head and neck squamous cancer (HNSC), we rebuild the functional networks linking specific TIME driver alterations to the TIME state they associate with.

**Results:**

The 477 TIME drivers that we identify are multifunctional genes whose alterations are selected early in cancer evolution and recur across and within cancer types. Tumour suppressors and oncogenes have an opposite effect on the TIME and the overall anti-tumour TIME driver burden is predictive of response to immunotherapy. TIME driver alterations predict the immune profiles of HNSC molecular subtypes, and perturbations in keratinization, apoptosis and interferon signalling underpin specific driver-TIME interactions.

**Conclusions:**

Overall, our study delivers a comprehensive resource of TIME drivers, gives mechanistic insights into their immune-regulatory role, and provides an additional framework for patient prioritisation to immunotherapy. The full list of TIME drivers and associated properties are available at http://www.network-cancer-genes.org.

**Supplementary Information:**

The online version contains supplementary material available at 10.1186/s13073-023-01197-0.

## Background

Cancer evolves within a stromal microenvironment with whom it engages in a dynamic crosstalk whereby genetic alterations in the cancer cells modulate the microenvironment and, in turn, the microenvironment sculpts the cancer genome [1–3]. Besides shaping cancer evolution, tumour-stroma interactions, especially with the tumour immune microenvironment (TIME), impact on overall prognosis and response to treatments including immunotherapy [4, 5]. Unravelling cancer-TIME interactions is therefore crucial to fully understand cancer biology.

Tumour-TIME interactions often involve genes that drive cancer evolution (cancer drivers). For example, loss-of-function (LoF) alterations in *TP53* reduce the anti-tumour infiltration of natural killer (NK) cells [6] while gain-of-function (GoF) alterations in *KRAS* promote pro-tumour infiltration of myeloid-derived suppressor cells [7]. Moreover, deregulations of the WNT and PI3K-AKT cancer pathways result in CD8^+^ T cell exclusion [8] and regulatory T cells increase [9], respectively.

Recently, systematic genetic screens have expanded the repertoire of genes that can modulate cancer immune response. A preferential loss of tumour suppressors has been observed in mice with a functional immune system where they likely promote immune escape [10]. Moreover, genome-wide CRISPR screens in co-cultures of cancer and cytotoxic T cells have identified gene losses conferring resistance to T cell-mediated killing [11]. Although these screens enable identification of TIME-interacting genes beyond cancer drivers, they rely on cell or animal models rather than human samples and have so far assessed the TIME role of LoF alterations neglecting that of GoF alterations.

Large cancer genomic and transcriptomic datasets allow to compute tumour-TIME associations in pan-cancer cohorts and are unbiased towards the alteration type. These studies have reported a prevalence of *PDL1* amplifications in immune-hot tumours [12, 13] as opposed to a high occurrence of *APC*, *KRAS*, *IDH1* or *FGFR3* mutations in immune-cold tumours [12–15]. They have so far focused mostly on anti-tumour immunity relying on the same list of drivers applied to the whole pan-cancer cohort. However, only very few drivers are shared across cancers and the absence of any further filtering on the actual cancer-specificity of the driver activity likely led to false-positive associations. Moreover, very little is still known about the molecular mechanisms of the tumour-TIME associations.

To overcome these limitations, here we have computed the interactions between manually curated and cancer-specific lists of drivers and five anti- and pro-tumour immune features of 6921 samples in 32 solid cancers. We have then investigated the properties of the resulting genes and their potential to predict response to immunotherapy. Taking head and neck squamous cancer (HNSC) as an example, we have rebuilt the tumour-TIME functional networks in the three HNSC subtypes. This enabled us to unravel the mechanisms linking driver alterations to the TIME modifications at the individual sample level.

## Methods

### Sample cohorts

A dataset of 7730 samples with quality-controlled mutation (single nucleotide variants, (SNVs) and indels), copy number and gene expression data in 32 solid cancer types from The Cancer Genome Atlas (TCGA) was assembled from the Genomic Data Commons portal (GDC, https://portal.gdc.cancer.gov/). Oesophageal cancer was divided into squamous cell carcinoma (OSCC) and adenocarcinoma (OAC) using TCGA annotation. Colon adenocarcinoma (COAD) was split into COAD-MSS and COAD-MSI according to the level of microsatellite instability (MSI) [16]. These samples were used to identify and characterise pan-cancer TIME drivers.

For the analysis of HNSC TIME drivers and their TIME drivers – TIME TFs functional networks, a dataset of 109 HNSC samples from the Clinical Proteomic Tumour Analysis Consortium (CPTAC) [17] with mutation, copy number and gene expression data was downloaded from GDC. These samples were added to the TCGA HNSC cohort for a total of 562 HNSCs.

### Data curation

SNVs and indels were annotated with ANNOVAR [18] (April 2018) and dbNSFP [19] v3. 0 and only those identified as damaging were retained. These included truncating (stopgain, stoploss, frameshift) mutations, hotspot mutations, missense mutations and splicing mutations predicted as damaging as described in [20]. Copy Number Alteration (CNA) segments, sample ploidy and sample purity were obtained using ASCAT [21] v. 2. 5. 2.  CNA segments were intersected with the exonic coordinates of 19,641 unique human genes in hg19 and CNA genes were identified as those with ≥ 25% of transcript length was covered by a CNA segment. RNA-Seq data were used to filter out CNAs with no effect on gene expression. Damaging gene gains were defined as CNA >2 times sample ploidy and significantly higher gene expression as compared to baseline expression. Expression distributions were compared using a Wilcoxon rank-sum test and corrected for multiple testing using Benjamini–Hochberg correction. Only gene gains with false discovery rate (FDR) < 0.05 were retained. Homozygous gene losses were defined as copy number = 0 and expression values <1 FPKM over sample purity. Heterozygous gene losses were defined as copy number = 1 or 0 and expression values >1 FPKM over sample purity. This resulted in 2,163,756 redundant genes damaged in 7730 TCGA samples. Of these, 511,048 genes acquired LoF alterations (homozygous deletions, truncating, missense damaging, or splicing mutations, double hits), while 1,652,708 genes were considered to acquire GoF alterations (hotspot mutations or damaging gene gains).

For the CPTAC cohort, damaging SNVs and indels were identified as described above. CNAs were derived using AscatNGS [22]. Sample ploidy was calculated as the average copy number of all segments weighted by segment length [22]. Sample purity was measured from gene expression data using ESTIMATE [23]. Gene gains, heterozygous and homozygous gene losses were defined as described above. In total, 26,450 redundant damaged genes were identified, for a total of 7891 LoF and 18,559 GoF alterations, respectively.

### Driver annotation

**Fig. 1:**
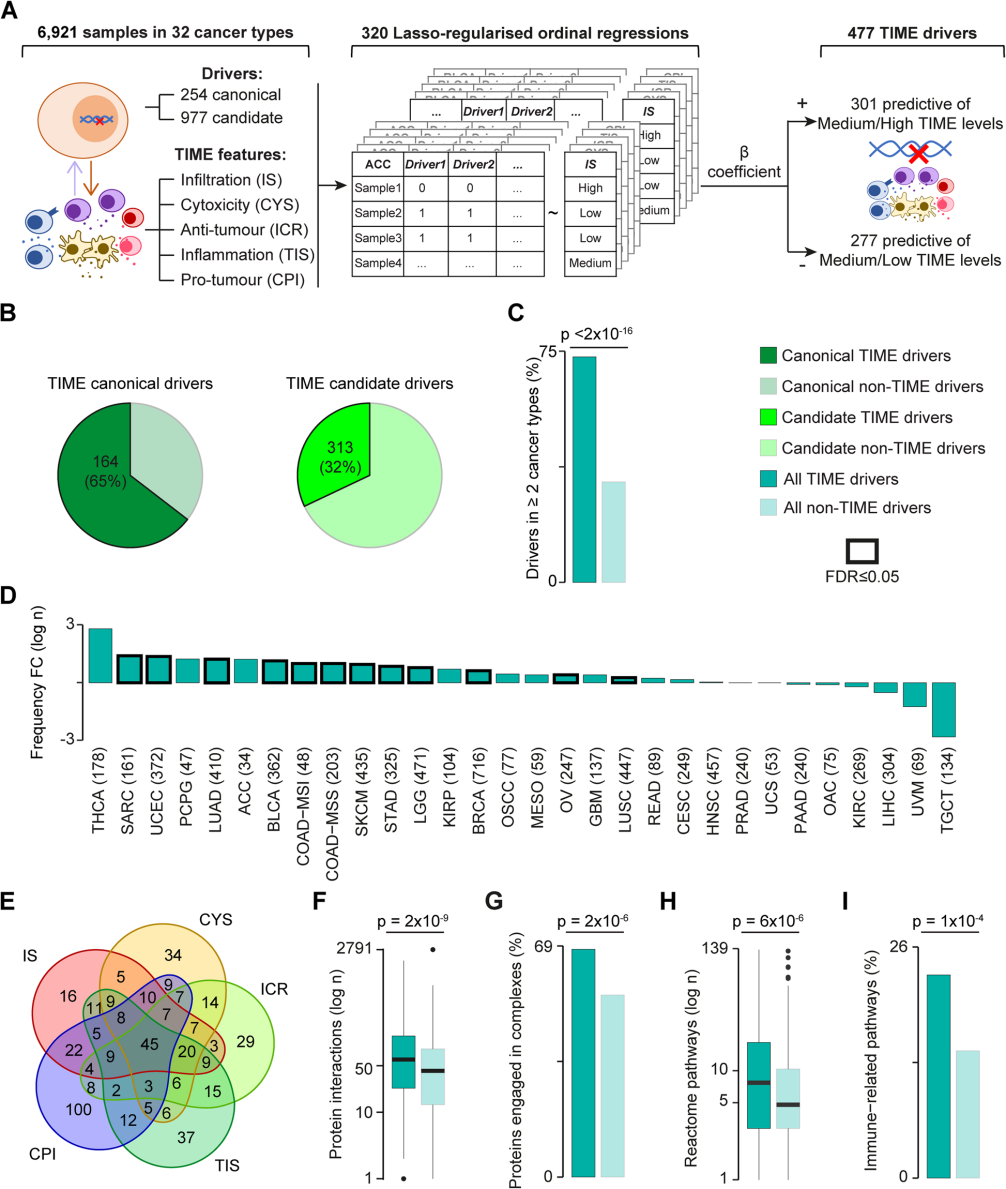
Identification and properties of TIME drivers. **A** Approach for TIME driver identification. Interactions between damaging alterations in cancer-specific drivers and TIME features were assessed in 32 TCGA cancer types using Lasso-regularised ordinal regression. Regressions were computed separately for canonical and candidate drivers and TIME features in each cancer type. $\beta >$ 0 and $\beta <$ 0 indicated that altered cancer drivers were predictive of medium/high or medium/low TIME levels, respectively. **B** Proportions of TIME canonical and candidate drivers over all drivers. **C** Proportions of TIME and non-TIME drivers in ≥ 2 cancer types. **D** Fold change (FC) of median frequency alterations of TIME and non-TIME drivers in each cancer type. The number of samples in each cancer type is shown in brackets. **E** Venn diagram of TIME drivers predictive of the five TIME features. **F** Distributions of protein–protein interactions in TIME and non-TIME drivers. **G** Proportions of TIME and non-TIME drivers that are part of protein complexes. **H** Distributions of level 2–9 Reactome pathways [39] containing TIME and non-TIME drivers. **I** Proportion of TIME and non-TIME drivers mapping to immune-related pathways as derived from MSigDB [40] and Reactome [39]. CPI, cancer-promoting inflammation; CYS, cytotoxicity score; FDR, false discovery rate; ICR, immunologic constant of rejection; IS, immune score; TIS, tumour inflammation signature. TCGA abbreviations are listed in Additional File 2: Table S1. Proportions (**C**,** G**,** I**) and distributions (**D**, **F**, **H**) were compared using Fisher’s exact test and Wilcoxon rank-sum test, respectively. In **D**, Benjamini–Hochberg correction for multiple testing was applied

The cancer drivers for each of 32 TCGA cancer types were retrieved from the Network of Cancer Genes (NCG, http://www.network-cancer-genes.org), which collects preferentially mutational drivers [20]. To add drivers altered through CNAs, focal amplifications and deletions in each cancer type were gathered from the FireBrowse portal (http://firebrowse.org) [24]. Amplification and deletion segments were intersected with 256 canonical and 1,405 candidate oncogenes and 254 canonical and 1318 candidate tumour suppressors [20], respectively. Drivers that fell for at least 25% of their transcript length within a CNA event were considered CNA drivers. Only tumour suppressors with LoF alterations and oncogenes with GoF alterations were further retained. Both LoF and GoF alterations were considered for drivers with unclassified roles. Finally, only drivers damaged in ≥ 2% or five samples were retained. In total 1231 (254 canonical and 977 candidate drivers, Fig. 1 and S1A) damaged in 6921 samples were used for the identification of TIME drivers.

The clonality of 27,763 damaging mutations affecting 1,231 drivers was measured using the cancer cell fraction (CCF) as described in [25]. Briefly, for each damaging mutation, the probability to have a CCF from 0. 01 to 1 with 0. 01 increments was calculated given the observed variant allele frequency (VAF), mutation copy number status, sample purity and normal copy number. The CCF with the highest probability was selected with the associated 95% confidence interval (CI). A damaging mutation was considered clonal if 95% CI of the CCF overlapped with 1; otherwise, it was considered subclonal. A driver was considered clonal when it had at least one clonal-damaging mutation.

The clonality of 38,846 damaging CNAs affecting 1,231 drivers was assessed with ABSOLUTE [26], using mutation VAFs and SNP6 array segmentation values obtained from GDC. ABSOLUTE was run with default parameters and using the cancer type-specific karyotype models for 6900 TCGA samples. Even in this case, the returned CCF with the highest probability was selected with the associated 95% CI. A CNA driver was considered clonal if a 95% CI of its CCF overlapped with 1; otherwise, it was considered subclonal.

### TIME features

To assess the cytotoxic anti-tumour infiltration score (CYS), 6445 samples were grouped into six clusters ordered from the lowest (CYS1) to the highest (CYS6) CYS levels, as defined in the original publication [12]. The six CYS levels were grouped into low (CYS1, 2), medium (CYS 3, 4) and high (CYS 5, 6) groups for consistency with the other features. To assess the immunologic constant of rejection (ICR), 6528 samples were grouped into low, medium or high ICR levels based on the expression distribution of 20 genes encoding IFN-simulated, regulatory, and effector immune molecules [15]. To assess the tumour inflammation signature (TIS), 6266 samples were grouped into low, medium or high TIS levels based on the signature values derived from the expression of 18 genes measuring adaptive immune response [27]. Overall immune infiltration (immune score, IS) and cancer-promoting inflammation (CPI) values were calculated for 6921 and 6728 samples, respectively, based on the expression of 141 genes using ESTIMATE [23] and as the mean of the Log_2_-transformed expression [28] of ten genes encoding known mediators of cancer-promoting inflammation [29], respectively. For IS, FPKM gene expression data from GDC were used to assess gene expression levels. For CPI, RSEM gene expression data from cBioPortal [30, 31] were used. Cancer samples were grouped into discrete categorical levels starting from the lowest (low) to the highest quartile (high) and assigning all remaining samples to the group with medium levels. The sample grouping was performed for each cancer type separately.

### Lasso-regularised ordinal regression

Lasso-regularised ordinal regression was used to estimate the probability of a given damaged driver to predict the TIME ordinal level of a given cancer sample using the glmnetcr function from the glmnetcr R package [32] v. 1. 0. 6. Input data consisted of a binary matrix whose rows corresponded to samples (observations) and columns to the driver alteration status (variable). TIME levels were encoded as ordered factor vectors with a size equal to the number of samples. Regression analysis was run for each TIME feature in each cancer type, considering canonical and candidate drivers separately and only samples with ≥ 1 damaged driver, for a total of 320 glmnetcr calls to fit 320 regression models. All analyses were run without variable standardisation and all other parameters were set to default values. In each regression, multiple steps of the model with the different values of lambda were ran and models with the minimum Akaike information criterion were used to extract non-zero $\beta$ coefficients [32].

### Protein–protein interaction and functional analysis

The number of non-redundant protein–protein interactions for TIME and non-TIME drivers were computed from BioGRID [33] v. 3. 5. 185, IntAct [34] v. 4. 2. 14, DIP [35] (February 2018), HPRD [36] v. 9 and Bioplex [37] v. 3. 0 as described in [20] and compared using a Wilcoxon rank-sum test. The proportion of proteins encoded by TIME and non-TIME cancer drivers that engage in complexes were derived from CORUM [38] v. 3. 0, HPRD [36] v. 9 and Reactome [39] v. 72 as described in [20] and compared using Fisher’s exact test.

Reactome [39] v. 72 level 2–9 pathways were used to calculate the numbers of pathways each of 821 drivers present in Reactome mapped to. These were compared between 335 TIME and 486 non-TIME cancer drivers using a Wilcoxon rank-sum test. A list of 2519 immune-related genes was derived combining genes mapping to the immune system level 1 pathway of Reactome [39] v. 72 and the immune-related pathways in MSigDB [40]. The proportions of immune-related TIME and non-TIME drivers were compared using a Fisher’s exact test.

### HNSC analysis

Human papillomavirus negative (HPV^−^) HNSCs were divided into CNA^high^ and CNA^low^ subtypes as described in [41] using a cohort of 1495 squamous cell carcinomas that included 1386 TCGA samples and 109 CPTAC HNSCs. CNA GISTIC2 loci were obtained from [41] for the TCGA cohort and from LinkedOmics [42] for the CPTAC HNSCs. Loci were classified as copy number neutral, low, and high CNAs and grouped with hierarchical clustering using Euclidean distance. Two clusters, one with 143 HPV^−^ CNA^low^ HNSCs and the other with 351 HPV^−^ CNA^high^ HNSCs were identified. TCGA classification overlapped with that in the original publication [41] for 94% of samples (Additional File 1: Figure S3A). Survival analysis was performed for 557 patients with available clinical data using the survminer R package v. 0. 4. 9 and compared between HNSC subtypes using the log-rank test.

CIBERSORTx [43] was run on the FPKM-normalised RNA-Seq data of 562 HNSCs using the LM22 signature to estimate the absolute abundance level of 22 immune populations. Absolute abundance scores were compared between HNSC subtypes using a Wilcoxon rank-sum test and corrected with Benjamini–Hochberg correction.

Since only FPKM gene expression data were available for all 562 HNSCs, the five TIME features were recalculated using FPKM instead of RSEM values, verifying that the two measures correlated positively (Additional File 1: Figure S3B-F). For CYS and ICR, clustering was done as described in the original publications [12, 15]. For TIS the same clustering strategy as for the TCGA cohort was applied. For IS and CPI, the score was calculated as described above.

For each TIME feature, the normalised sample proportion of a HNSC subtype $i$ in a TIME level $j$ was calculated as:

$$(\frac{nij}{ni})/(\frac{Nj}{N})$$

where ${n}_{ij}$ is the number of HNSCs in subtype $i$ with TIME level $j$; ${n}_{i}$ is the total number of HNSCs in subtype $i$; ${N}_{j}$ is the number of all HNSCs with TIME level $j$; and N is the total number of HNSCs.

### TIME drivers – TIME TFs functional network

A list of 1471 genes annotated with the GO:0006355 term (regulation of DNA-templated transcription) of Gene Ontology (release 2022–05) [44, 45] was considered *bona fide* transcription factors (TFs) and used as input for ARACNE-AP [46] together with the gene expression profiles of 562 HNSCs with default parameters (Fig. 4A). The resulting HNSC transcriptional regulatory network composed of 1443 TFs, 18,067 targets and 202,512 interactions was used to infer the sample-level TF protein activity using VIPER [47], resulting in 1211 HNSC-active TFs.

The expression levels of downstream targets of each active TFs were compared between HNSC subtypes and 100 adjacent normal tissues using ms-VIPER [47]. Overall, 271, 113 and 212 differentially active (DA) TFs (*p*-value < 0. 05) were found in HPV^+^, HPV^−^ CNA^low^ and HPV^−^ CNA^high^ HNSCs, respectively, for a total of 398 unique DA TFs. Pearson’s correlations were calculated between the protein activity of each DA TF and each TIME feature to retrieve the DA TFs that significantly correlated (FDR < 0. 1) with TIME in each HNSC subtype (TIME TFs). Overall, 51, 103 and 159 TIME TFs were found in HPV^+^, HPV^−^ CNA^low^ and HPV^−^ CNA^high^ HNSCs, respectively, for a total of 240 unique DA TFs.

TIME TFs were tested for statistical association with the 53 HNSC TIME drivers, comparing their protein activity in HNSCs with and without TIME driver alterations using the Wilcoxon rank sum test. Overall, 131, 373 and 882 TIME TF- TIME driver significant associations (FDR < 0. 1) were found in HPV^+^, HPV^−^ CNA^low^ and HPV^−^ CNA^high^ HNSC, respectively, for a total of 1386 associations. TieDIE [48] was applied to find functional interactions between significantly associated TIME TFs and HNSC TIME drivers. The prior knowledge network (PKN) for TieDIE was assembled from 542,397 protein–protein [20], 12,730 phosphorylation [49], 15,104 genetic [33] and 34,877 signalling interactions [50] across 18,053 human genes. Fourteen TIME drivers–TIME TFs functional networks were rebuilt in each HNSC subtype and TIME feature, seven of which had an influence score significantly higher (*p*-value < 0. 08) than random networks with the same degree distribution (Additional File 2: Table S11). Starting from these networks, coherent subnetworks were defined as those with maximum three nodes between the TIME driver and the TIME TF and a positive coherency score [48] (Additional File 2: Table S12). TIME TF targets were functionally annotated using pathway enrichment analysis as described in [51] (Additional File 2: Table S13). GSEA [52] v4. 3. 2 was used with a gene set permutation test of 1,000 iterations.

## Results

### TIME drivers are multifunctional genes commonly altered across cancers

To identify the cancer genes interacting with specific states of the TIME (TIME drivers), we derived a reliable set of genes specifically contributing to the evolution of each of the 32 TCGA cancer types (Additional File 1: Figure S1A). We started from a pan-cancer collection of experimentally validated (canonical) and computationally predicted (candidate) drivers [20] and assigned them to each cancer type according to an expert annotation of the literature. We then retained only drivers with damaging alterations in 7730 TCGA samples with matched genomic and transcriptomic data. We considered LoF alterations in tumour suppressors, GoF alterations in oncogenes, and both types of alterations in drivers with unclassified roles. We removed rarely damaged drivers for which no reliable interaction with the TIME could be computed. The final list was composed of 254 canonical and 977 candidate drivers with damaging alterations in 6921 samples across the 32 cancer types (Fig. 1A, Additional File 2: Table S1, Table S2).

To characterise the TIME features of these tumours, we used five gene expression signatures indicative of overall tumour immune infiltration (IS) [23], cytotoxic anti-tumour infiltration (CYS) [12], anti-tumour T-helper activity (ICR) [15], anti-tumour inflammation state (TIS) [27], and cancer-promoting inflammation (CPI) [28, 29] (Additional File 2: Table S3). The five gene signatures had a minimal overlap (Additional File 1: Figure S1B), confirming that they captured distinct TIME properties. Since IS and CPI were available only for a subset of samples, we re-computed them for the whole cohort, verifying that our results reproduced those previously published [23, 28, 29] (Additional File 1: Figure S1C, D).

We grouped samples into low, medium, and high TIME levels of each TIME feature based on the corresponding score distribution in each cancer type. We used categorical values rather than the original scores to make the analysis more interpretable and comparable across features and cancer types. We then calculated the probability of a cancer driver to predict the TIME level of the sample where it was altered using ordinal logistic regression with Lasso regularisation for each feature in each cancer type (Fig. 1A). Driver-TIME feature pairs with a positive $\beta$ regression coefficient indicated that samples with damaging alterations in that driver were likely to have medium or high levels for that TIME feature. Driver-TIME features pairs with a negative $\beta$ regression coefficients indicated the opposite.

Overall, we identified 477 TIME drivers whose damaging alterations predicted higher (301) or lower (277) TIME levels in 30 cancer types (Fig. 1A, Additional File 2: Table S4, available at http://www.network-cancer-genes.org). These predictions were enriched in experimentally validated TIME drivers (66 of 102, *p* = 4 × 10^−8^, Fisher’s exact test, Additional File 2: Table S5), supporting the robustness of our approach. Cholangiocarcinoma and kidney chromophobe cancer did not show any significant interaction, possibly because of the low statistical power due to the small sample size. Predicted TIME drivers included 164 canonical and 313 candidate drivers, indicating that most well-known cancer drivers can interfere with the immune system (Fig. 1B). Unexpectedly since cancer drivers tend to be cancer-specific [20], TIME drivers were instead recurrently damaged across cancer types (Fig. 1C) and samples (Fig. 1D, Additional File 2: Table S6). Moreover, more than 40% of them were predicted as TIME drivers in multiple cancer types (Additional File 2: Table S4). They included well-known TIME drivers such as *TP53*, *PTEN*, *ARID1A*, and *KRAS*, but also *PIK3CA*, *CDKN2A*, or *TERT* for which no or very little interactions with the TIME have been described. Most TIME drivers (261, 55%) were predictive of at least two features, and 45 of all five of them (Fig. 1E). An example was *BRAF*, whose V600E mutation is highly immunogenic [53], despite BRAF signalling promoting pro-tumour inflammation [54].

Our results depicted TIME drivers as genes recurrently damaged across cancer samples and types and able to interact plastically with multiple TIME features. This suggested that TIME drivers were likely multifunctional genes involved in several biological processes. To test this hypothesis, we computed the number of interactions of TIME drivers in the protein–protein interaction network. We confirmed that TIME drivers encoded proteins engaging in a significantly higher number of protein–protein interactions (Fig. 1F) and protein complexes (Fig. 1G) compared to non-TIME drivers. Moreover, TIME drivers mapped to a significantly higher number of biological pathways (Fig. 1H) and were involved in a higher number of immune-related functions (Fig. 1I), confirming that they are multifunctional genes.

### TIME tumour suppressors and oncogenes predict opposite TIME states

**Fig. 2:**
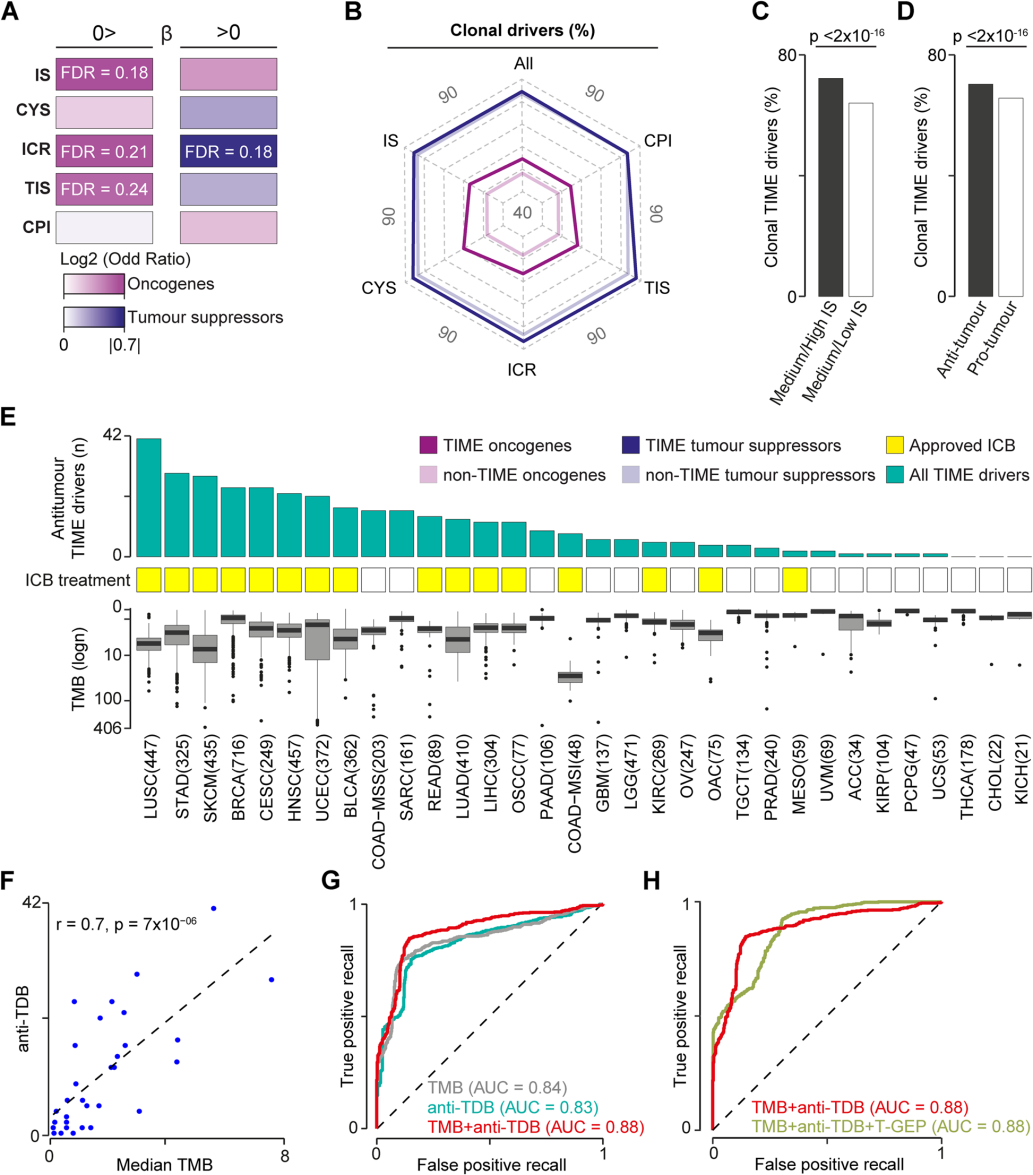
Effects of tumour suppressors and oncogenes on TIME and ICB response. **A** Enrichment of TIME drivers predictive of medium/low ($\beta <0)$ or medium/high ($\beta >0)$ TIME levels in tumours suppressors and oncogenes. **B** Proportions of TIME and non-TIME tumour suppressors or oncogenes with clonal damaging alterations. **C** Proportions of clonal TIME drivers predictive of high or low immune infiltration. **D** Proportions of clonal TIME drivers predictive of an anti-tumour (CYS, TIS, ICR $\beta >0$, or CPI $\beta$ < 0) and pro-tumour (CYS, TIS, ICR $\beta <0$, or CPI $\beta$ > 0) TIME. **E** Number of antitumour TIME drivers, approval ICB treatment and TMB across cancer types. The number of samples for each cancer type is shown brackets. **F** Pearson’s correlation between the median TMB and the number of antitumour TIME drivers in 31 cancer types, excluding COAD-MSI. ROC curves comparing the performance of TMB and anti-TDB (**G**) and T-cell-inflamed GEP (**H**) in predicting response to ICB. Recall rates and AUCs were calculated across 100 cross-validations. AUC, area under the curve; CPI, cancer-promoting inflammation; CYS, cytotoxicity score; ICB, immune checkpoint blockade; ICR, immunologic constant of rejection; IS, immune score; anti-TDB, antitumour TIME driver burden; GEP, gene expression profile; TIME, tumour immune microenvironment; TIS, tumour inflammation signature; TMB, tumour mutational burden; TOB, TIME oncogene burden; TTB, TIME tumour suppressor burden. TCGA abbreviations are listed in Additional File 2: Table S1. Proportions (**A–D**) were compared using Fisher’s exact test. In **A**, Benjamini–Hochberg correction for multiple testing was applied

Given the different modes of action, we sought to analyse the TIME interactions of tumour suppressors and oncogenes separately. Overall, we found that their alterations had an opposite effect on the TIME composition. While tumour suppressors were enriched in TIME drivers predictive of a hot anti-tumour TIME, oncogenes were enriched in TIME drivers predictive of a cold pro-tumour TIME (Fig. 2A, Additional File 2: Table S7). These observations suggested that tumour suppressor alterations preferentially helped tumours to survive in a hot TIME. Oncogene alterations, instead, sustained tumour growth in the presence of a pro-tumour TIME or directly inhibited anti-tumour TIME.

We reasoned that, if alterations in TIME tumour suppressors favoured immune escape, they were likely to occur early in tumour evolution. To test this hypothesis, we computed the proportion of clonal drivers and found that TIME tumour suppressors were enriched in clonal drivers compared to TIME oncogenes and non-TIME tumour suppressors (Fig. 2B, Additional File 2: Table S8). Moreover, the proportion of clonal TIME drivers predictive of high immune infiltration was significantly higher than that of TIME drivers predictive of low immune infiltration (Fig. 2C). Similarly, anti-tumour TIME drivers were enriched in clonal alterations compared to pro-tumour TIME drivers (Fig. 2D). Therefore, cancers with a hot TIME select alterations in tumour suppressors very early in their evolution. Interestingly, also the proportion of TIME oncogenes with clonal alterations was significantly higher than that of non-TIME oncogenes (Fig. 2B, Additional File 2: Table S8). This suggested that, independently on their effect, drivers involved in the tumour-immune interactions are altered earlier than other drivers. Interestingly, 74% of genes driving somatic clonal expansion in non-cancer tissues [55] were TIME drivers, indicating that their interaction with the immune system may even predate cancer transformation.

A hot TIME is needed for an effective response to immune checkpoint blockade (ICB) [4]. We therefore hypothesised that the number of antitumour TIME drivers in a cancer type (i.e. its antitumour TIME driver burden, anti-TDB) could predict its response to ICB. To test this hypothesis, we considered whether ICB treatment had been approved for that cancer type [56, 57] and used the median tumour mutational burden (TMB) for comparison. Unsurprisingly since both anti-TDB and TMB depend on the overall number of cancer alterations, they were positively correlated (Fig. 2E, F). We used Bayesian logistic regression to account for this correlation and avoid overfitting [58]. Moreover, we tested maximum three variables at a time resulting in at least ten observations per variable to further minimise overfitting [59, 60]. TMB and anti-TDB showed comparable predictive power (*p* = 0. 003, Additional File 1: Figure S2A). We therefore compared their predictive power alone or in combination using Receiver Operating Characteristic (ROC) curves. We confirmed that TMB and anti-TDB alone were significant predictors of response, but their combination further improved the predictive power (Fig. 2G). In line with their antitumour TIME interactions, antitumour TIME tumour suppressor burden (anti-TTB) had higher predictive power than the TIME oncogene burden (anti-TOB, Additional File 1: Figure S2B). Moreover, no significant difference in prediction accuracy could be observed between training and test datasets (Additional File 1: Figure S2C), confirming that the model was not overfitted.

In a similar way, we tested other described predictors of response to ICB, namely T-cell-inflamed gene-expression profile (GEP) [61], *PDL1* [62], *CD8A* [63] and *CXCL9* [64] expression levels, gender [65] and age at diagnosis [66]. Of those, only T-cell-inflamed GEP, *CD8A* and *CXCL9* expression showed significant predictive power across all cancer types (Additional File 1: Figure S2A). Moreover, T-cell-inflamed GEP, *CD8A* and *CXCL9* expression were positively correlated (Additional File 1: Figure S2D-F) and T-cell-inflamed GEP showed the highest predictive power among the three (Additional File 1: Figure S2A). Therefore, we decided to test the predictive power of T-cell-inflamed GEP when added to TMB and anti-TDB. However, the addition of T-cell-inflamed GEP did not further improve the predictive power compared to when TMB and anti-TDB were analysed together (Fig. 2H).

### TIME drivers predict the TIME profiles of head and neck cancer subtypes

To gain further insights into driver-TIME interactions, we focused on head and neck squamous cell carcinoma (HNSC), a group of genetically heterogeneous cancers from multiple anatomical sites [67]. The two main subtypes of HNSC are caused by human papillomavirus (HPV^+^ HNSC) and cigarette smoking (HPV^−^ HNSC), respectively. HPV^+^ tumours show less genetic instability, respond better to treatment, and have an overall better prognosis [68]. Despite having among the highest leukocyte infiltration [14], HNSC shows variable response to immunotherapy [56]. This makes it an interesting cancer type to further investigate the dynamic of driver-TIME interactions.

**Fig. 3:**
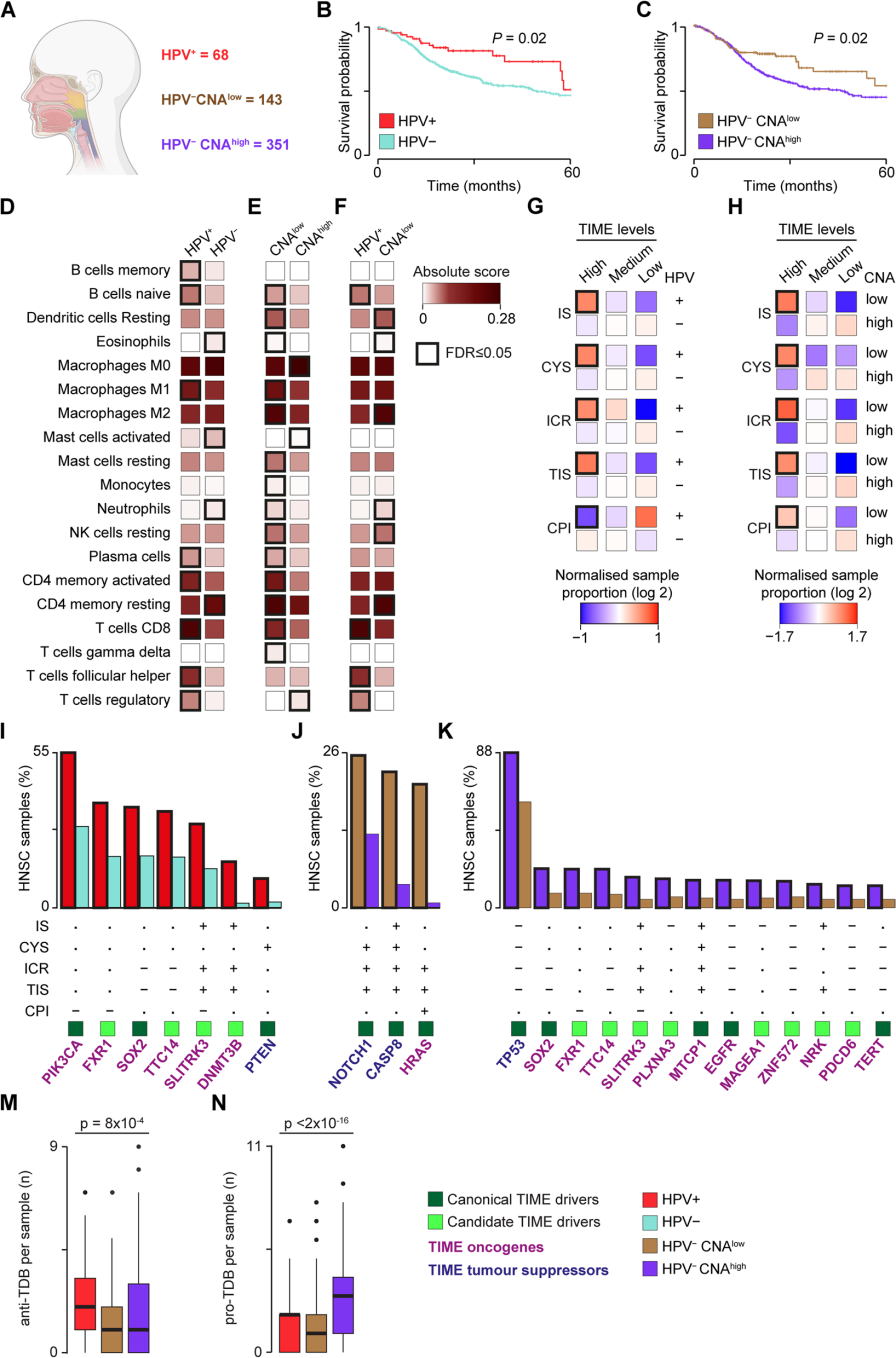
Immune profiles and TIME driver alterations of HNSC. **A** HNSC extended cohort. HNSCs collected from TCGA and CPTAC were divided in HPV^+^, HPV^−^ CNA^low^ and HPV^−^ CNA^high^ samples based on HPV infection and level of aneuploidy [41]. Kaplan–Meier survival curves between HPV^+^ and HPV^−^ (**B**) or HPV^−^ CNA^low^ and HPV^−^ CNA^high^ (**C**) HNSC patients. Overall survivals were compared using the log-rank test. Comparison of CIBERSORTx absolute score medians between HPV^+^ and HPV^−^ (**D**); HPV^−^ CNA^low^ and HPV^−^ CNA^high^ (**E**); or HPV^+^ and HPV^−^ CNA^low^ (**F**) HNSCs. Only immune cell types enriched in at least one HNSC subtype are shown. Comparison of sample proportion in the five TIME features between HPV^+^ and HPV^−^ (**G**) or HPV^−^ CNA^low^ and HPV^−^ CNA^high^ (**H**) HNSCs (see Methods). TIME drivers more frequently damaged in HPV^+^ HNSCs (**I**), HPV^−^ CNA^low^ (**J**), or HPV^−^ CNA^high^ HNSC samples (**K**). For HPV^−^ CNA^high^ HNSCs only the top 13 TIME drivers are shown (full list in Additional File 2: Table S4). CPI = cancer-promoting inflammation; CYS, cytotoxicity score; CPTAC, Clinical Proteomic Tumour Analysis Consortium; FDR, false discovery rate; HPV, human papillomavirus; ICR, immunologic constant of rejection; IS, immune score; TIS, tumour inflammation signature. Proportions were compared using Fisher’s exact test (**D–F**,** I–K**) or Mantel–Haenszel chi-square test (**G**,** H**). Distributions (**M**,** N**) were compared using Kruskal − Wallis test. In **D–K**, Benjamini–Hochberg correction for multiple testing was applied

We expanded the TCGA HNSC cohort to include samples from the Clinical Proteomic Tumour Analysis Consortium (CPTAC) [17], for a total of 562 HNSCs with matched genomic and transcriptomic data (Fig. 3A). Of these, 68 were HPV^+^ HNSC. Based on copy number alterations (CNAs) [41], we further divided the remaining HPV^−^ HNSCs into 351 CNA^high^ and 143 CNA^low^ samples (Fig. 3A, Additional File 1: Figure S3A). We confirmed that HPV^+^ HNSC patients have better overall survival (Fig. 3B) and, within the HPV^−^ group, high levels of aneuploidy confer worse prognosis [67] (Fig. 3C).

We quantified the immune infiltrates in samples of the three HNSC subtypes using their gene expression profiles and confirmed the hot anti-tumour TIME of HPV^+^ HNSCs (Fig. 3D, Additional File 2: Table S9). Interestingly, we observed an overall higher anti-tumour immunity in HPV^−^ CNA^low^ compared to HPV^−^ CNA^high^ HNSCs (Fig. 3E). HPV^−^ CNA^high^ HNSCs were instead enriched in M0 macrophages and regulatory T cells, suggesting that these pro-tumour immune infiltrates could contribute to their worse prognosis. When compared directly, HPV^+^ and CNA^low^ HNSCs showed a different immune infiltration profile, with the former enriched in T cells but depleted in NK cells and neutrophils compared to the latter (Fig. 3F). To test whether these differences were reflected in the five TIME features, we grouped the 562 HNSCs into low, medium, and high TIME levels in each TIME feature according to the corresponding distribution. We then compared the proportion of samples of the three HNSC subtypes in each TIME level. HPV^+^ samples consistently showed high proportion of samples with high and medium anti-tumour TIME features (Fig. 3G, Additional File 2: Table S10), confirming that they are immune-hotter than HPV^−^ HNSCs. Similarly, HPV^−^ CNA^low^ HNSCs confirmed to be immune-hotter than HPV^−^ CNA^higher^ HNSCs (Fig. 3H, Additional File 2: Table S10).

To test whether the TIME features of the three HNSC subtypes could be explained by their TIME driver alteration profile, we compared the frequency of the 53 HNSC TIME drivers across subtypes (Additional File 2: Table S4). Five of the seven TIME drivers significantly more frequently damaged in HPV^+^ HNSCs were predictive of high anti-tumour or low pro-tumour TIME (Fig. 3I). Similarly, all three TIME drivers more frequent in HPV^−^ CNA^low^ HNSCs were predictive of high anti-tumour immunity, while most TIME drivers more frequent in HPV^−^ CNA^high^ were predictive of low anti-tumour immunity (Fig. 3J, K). Moreover, HPV^+^ and HPV^−^ CNA^low^ HNSCs showed significantly higher anti-tumour or lower pro-tumour TDB per sample than HPV^−^ CNA^high^ HNSCs (Fig. 3M, N). These results indicated that the distinct immune profiles within HNSCs segregate with the distinct TIME driver alteration profiles across molecular subtypes.

### Functional networks uncover the molecular mechanisms of driver-TIME interactions

**Fig. 4:**
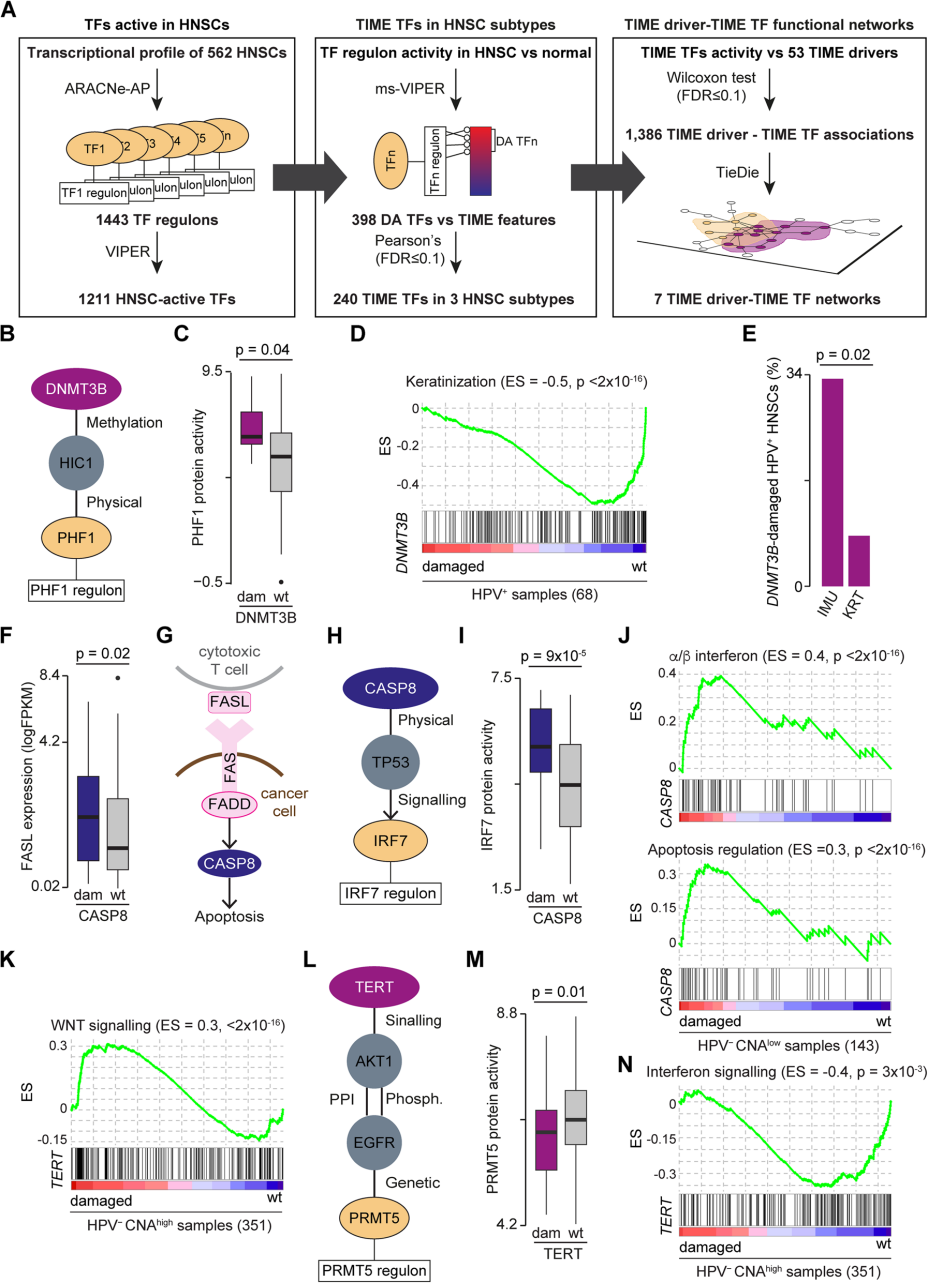
Driver–TIME functional networks in HNSC subtypes. **A** Reconstruction of HNSC driver-TIME functional networks. HNSC transcriptional regulatory network was used to identify the transcription factors (TFs) differentially active (DA) in the three HNSC subtypes that correlated with TIME features and were associated with TIME drivers. Combining functional data, the significant functional networks linking these drivers to TIME TFs were derived. **B** DNMT3B functional subnetwork in HPV^+^ HNSCs. **C** Comparison of PHF1 protein activity between *DNMT3B*-damaged and wild-type (wt) HPV^+^ HNSCs. **D** Gene set enrichment analysis (GSEA) plot comparing the activation of the keratinization pathway between *DNMT3B*-damaged and wt HPV^+^ HNSCs. **E** Comparison of *DNMT3B*-damaged samples between immune (IMU) and keratinization (KRT) HPV^+^ HNSCs from [72]. **F** Comparison of *FASL* gene expression levels between *CASP8*-damaged and wt HPV^−^ CNA^low^ HNSCs. **G** Schematic of cytotoxic T-cell induced apoptosis of cancer cells through the FAS-FASL cascade. **H** CASP8 functional subnetwork in HPV^−^ CNA^low^ HNSCs. **I** Comparison of IRF7 protein activity in *CASP8*-damaged and wt HPV^−^ CNA^low^ HNSCs. **J** GSEA plots comparing the activation of the α/β interferon signalling and apoptosis regulation pathways between *CASP8*-damaged and wt HPV^−^ CNA^low^ HNSCs. **K** GSEA plot comparing the activation of the WNT signalling pathway between *TERT-*damaged and wt HPV^−^ CNA^high^ HNSCs. **L** TERT functional subnetwork in HPV^−^ CNA^high^ HNSCs. **M** Comparison of PRMT5 protein activity between *TERT-*damaged and wt HPV^−^ CNA^high^ HNSCs. **N** GSEA plot comparing the activation of the interferon signalling pathway between *TERT*-damaged and wt HPV^−^ CNA^high^ HNSCs. CNA, copy number alteration; HNSC, head and neck squamous cell carcinoma; HPV, human papilloma virus; TIME, tumour immune microenvironment. Distributions (**C**, **F**, **I**, **M**) were compared using Wilcoxon rank-sum test. Proportions (**E**) were compared using Fisher’s exact test. GSEAs (**D**,** J**,** K**,** N**) were performed using gene set permutation tests

Regression models can reveal significant interactions between genetic drivers and the TIME but not the directionality of these interactions. To unravel the functional links between driver alterations and TIME features more directly, we rebuilt the transcriptional regulatory network of 1443 transcription factors (TFs) in 562 HNSCs using their expression profiles (Fig. 4A). Measuring the corresponding TF protein activity, we found 1211 TFs overall active in HNSC and 398 differentially active in the three HNSC subtypes. Of these, 240 showed a significant correlation with TIME features in one of the three HNSC subtypes (TIME TFs). Comparing the protein activity of these TIME TFs in HNSCs with and without damaging alterations in the 53 HNSC TIME drivers, we found 1386 TIME driver – TIME TF associations. We then combined several types of functional data (Methods) and identified seven functional networks linking HNSC TIME drivers and TIME TFs (Fig. 4A, Tables S10). Since these networks comprised between 37 and 203 functional nodes (Additional File 2: Table S12), we extracted the coherent subnetworks connecting TIME drivers to TIME TFs through maximum three nodes.

These subnetworks enabled investigation of the transcriptional programmes directly linking TIME driver alterations to the TIME features in each HNSC subtype. For example, the TIME oncogene *DNMT3B*, predictive of a hot TIME, was frequently damaged in HPV^+^ HNSCs (Fig. 3I). DNMT3B was part of the HPV^+^/TIS subnetwork involving HIC1 and the TIME TF PHF1 (Fig. 4B, Additional File 2: Table S12). DNMT3B is known to methylate HIC1 [69] inhibiting PHF1 recruitment and activating its transcriptional repression programme [70]. Consistently, we found a higher PHF1 protein activity (Fig. 4C) and a downregulation of keratinization (a pathway enriched in PHF1 targets, Additional File 2: Table S13) in *DNMT3B*-damaged HPV^+^ HNSCs (Fig. 4D). Keratinocytes have recently been reported to inhibit T cell proliferation by secreting T cell-modulating cytokines [71]. This could explain how *DNMT3B* amplification could lead to higher immune infiltration. Interestingly, HPV^+^ HNSCs have been divided into two transcriptional subtypes, one (HPV^+^ KRT) characterised by high keratinocyte differentiation and the other (HPV^+^ IMU) with a strong immune response [72]. Using the same dataset [72], we verified that *DNMT3B* was more frequently damaged in HPV^+^ IMU HNSCs (Fig. 4E). This independently supported the hot anti-tumour TIME induced by *DNMT3B* amplification.

Next, we investigated the TIME role of the tumour suppressor *CASP8* whose damaging alterations were predictive of anti-tumour immunity and were enriched in HPV^−^ CNA^low^ HNSCs (Fig. 3J). Two lines of evidence supported a role of *CASP8* loss in immune escape in HPV^−^ CNA^low^ HNSC. The first was that *FASL,* a cytotoxic T cell-induced trigger of apoptosis [73] was upregulated in *CASP8*-damaged HPV^−^ CNA^low^ HNSCs (Fig. 4F). Since CASP8 is the downstream target of the FASL-initiated apoptotic cascade (Fig. 4G), its loss could prevent cancer cells to undergo apoptosis. The second line of evidence came from the HPV^−^ CNA^low^ HNSC subnetworks where CASP8 interacts with the TIME TF IRF7 through TP53 (Fig. 4H, Additional File 2: Table S12). Since CASP8 loss stabilises TP53 [74], we expected higher IRF7 activity in *CASP8*-damaged HPV^−^ CNA^low^ HNSCs, which was indeed confirmed (Fig. 4I). IRF7 targets were enriched in several immune and apoptosis-related pathways (Additional File 2: Table S13). Accordingly, we found a significant upregulation of both a/b interferon signalling and apoptosis-negative control *CASP8*-damaged HPV^−^ CNA^low^ HNSCs (Fig. 4J). This further confirmed that apoptosis reduction was a CASP8-induced immune escape mechanism in HPV^−^ CNA^low^ HNSCs with a hot TIME.

Lastly, we analysed the functional network of the TIME oncogene *TERT*, predictive of a cold TIME whose GoF alterations were enriched in HPV^−^ CNA^high^ HNSCs (Fig. 3K). *TERT* has been reported to contribute to the WNT-$\beta$-catenin pathway through its interaction with the $\beta$-catenin transcriptional complex [75], although in a highly context-dependent manner [76]. In our data, HPV^−^ CNA^high^ HNSCs with damaging alterations in *TERT* showed significant WNT upregulation (Fig. 4K). WNT activation has been linked to immune exclusion [8], which could explain the role of TERT in inducing a cold TIME. Moreover, TERT was in the same HPV^−^ CNA^high^ subnetwork of the TIME TF PRMT5 (Fig. 4L, Additional File 2: Table S12). TERT activation is known to induce AKT1-mediated EGFR phosphorylation that, in turn, downregulates PRMT5 through a negative genetic interaction [77]. We confirmed a lower PRMT5 protein activity (Fig. 4M) and the downregulation of interferon signalling (Fig. 4N), one of the pathways enriched in PRMT5 targets (Additional File 2: Table S13) in HPV^−^ CNA^high^ HNSCs with damaging alterations in *TERT*. A lower interferon activity could reduce the production of T cell chemo-attractants resulting in a cold TIME.

## Discussion

In this study, we predicted the functional interactions between the genetic drivers of 6,921 cancers and their immune microenvironment. Despite the analysis being conducted separately in 30 cancer types, the predicted TIME drivers shared key properties, including high multifunctionality, plasticity in their interaction with the TIME, and recurrent damaging alterations across cancer types and samples. These properties support a multifaceted role of TIME drivers in promoting tumour evolution through both cancer-intrinsic and cancer-extrinsic mechanisms and suggest that they can interfere with multiple TIME features likely in a tissue-specific manner.

We found an enrichment of TIME tumour suppressors in early drivers and their alterations are predictive of a hot anti-tumour TIME. These observations strongly suggest that tumour suppressors are involved in immune evasion supporting the recently reported preferential loss of tumour suppressors in mice with a functional adaptive immunity [10]. Unlike tumour suppressors, TIME oncogenes were preferentially damaged in samples with a cold TIME, in line with the documented role of *MYC*, *HRAS* and *BRAF* oncogenes in inducing inflammatory chemokines and cytokines [78]. The opposite effect of tumour suppressors and oncogenes on the TIME could reflect their broad functional differences, with the former mostly involved in controlling cell cycle, DNA repair and apoptosis and the latter enriched in signalling genes [79]. In all cases, TIME alterations occur earlier than those of other drivers, suggesting that the interactions between the mutated epithelium and the immune cell compartment are likely to start well before the epithelial cells become fully transformed [55].

The burden of antitumour TIME drivers, particularly tumour suppressors, can predict whether a cancer type is responsive to ICB and improves the predictive power of TMB. The identification of patients who are most likely to benefit from ICB treatment is an open clinical question since response to ICB primarily assessed in the clinic and translational biomarkers are still lacking [80]. For example, although ICB treatment is standard of care in recurrent HNSC [81], the majority of patients will not respond [82] exposing them to unnecessary toxic effects and worse survival. In clinical practice, the combined positive score based on the number of PD-L1 positive cells over all tumour cells is used for eligibility to ICB treatment [83]. Still, only 30% of HNSC patients will respond [56]. We showed that HPV^+^ but also HPV^−^ CNA^low^ HNSCs tend to have a hot anti-tumour TIME while HPV^−^ CNA^high^ HNSCs are usually deprived of immune infiltration. This would suggest a prioritisation of ICB treatment only in patients with HPV^+^ and HPV^−^ CNA^low^ HNSC subtypes.

Unlike TMB that has a sample-specific value, anti-TDB is a feature of the cancer type and cannot predict response of the individual patient to ICB. To overcome this limitation and unravel the molecular mechanisms of the driver-TIME interactions, we rebuilt the transcription regulatory networks linking driver alterations to TIME states. We identified TIME-driver functional networks for 33 HNSC TIME drivers, indicating how alterations in these genes interfere with the TIME. For example, *DNMT3B*-damaged HPV^+^ HNSCs significantly overlap with the recently identified HPV^+^ IMU subtype, which shows better prognosis and high immune infiltration [72]. Our data show that this is likely achieved through a reduction of keratinocyte differentiation induced by *DNMT3B* amplification. Therefore, patients with *DNMT3B*-damaged HPV^+^ HNSCs are good candidates for a successful ICB treatment. Similarly, CASP8-induced immune escape through apoptotic inhibition is another mechanism evolved by HPV^−^ CNA^low^ HNSCs to survive a high anti-tumour infiltration. Our prediction is that also this subgroup of patients would benefit from ICB treatment. On the contrary, TERT activation modulates a cold TIME, suggesting that HPV^−^ CNA^high^ patients with this alteration would not benefit from immunotherapy.

## Conclusions

Our study provides a comprehensive set of driver-TIME interactions and mechanistic insights into their crosstalk. This could be further explored in experimental and clinical settings for the development of robust and cancer-specific biomarkers of response to immunotherapy.

## Supplementary Information

**Additional file 1:** **Figure S1.** Input preparation for TIME driver identification. **Figure S2.** Prediction of ICB response in 32 cancer types. **Figure S3.** CNA profile clustering of HNSC samples.

**Additional file 2: Table S1.** Cancer drivers used in the analysis. **Table S2.** Canonical and candidate drivers damaged in 7,730 TCGA samples. **Table S3.** Gene signatures used to compute TIME features. **Table S4.** Predicted TIME drivers using Lasso regularised ordinal regression. **Table S5.** True positive TIME drivers collected from the literature. **Table S6.** Frequency of TIME and non-TIME driver alterations per cancer type. **Table S7.** Proportion of oncogenes and tumour suppressors predictive of TIME levels. **Table S8.** Comparison of clonality between TIME and non-TIME drivers. **Table S9.** Literature support for TIME differences across HNSC subtypes. **Table S10.** Comparison of TIME levels between HNSC molecular subtypes. **Table S11.** TIME driver-TIME TF functional networks in HNSC subtypes. **Table S12.** Components of HNSC TIME drivers - TIME TFs functional networks. **Table S13.** Functional annotation of TIME TF gene targets in selected coherent subnetworks.

## Data Availability

The datasets supporting the conclusions of this article are included within the article and its additional files.

## Abbreviations

ACC: Adrenocortical carcinoma
Anti-TDB: Antitumour TIME driver burden
AUC: Area under the curve
BLCA: Bladder urothelial carcinoma
BRCA: Breast invasive carcinoma
CCF: Cancer cell fraction
CESC: Cervical squamous cell carcinoma and endocervical adenocarcinoma
CHOL: Cholangiocarcinoma
CI: Confidence interval
CNA: Copy number alteration
COAD-MSI: Colon adenocarcinoma microsatellite instable
COAD-MSS: Colon adenocarcinoma microsatellite stable
CPI: Cancer-promoting inflammation score
CPTAC: Clinical proteomic tumour analysis consortium
CYS: Cytotoxic score
DA: Differentially active
FDR: False discovery rate
GBM: Glioblastoma multiforme
GDC: Genomic data commons
GEP: Gene expression profile
GoF: Gain-of-function
HNSC: Head and neck squamous cell carcinoma
HPV: Human papilloma virus
ICB: Immune checkpoint blockade
ICR: Immunologic constant of rejection
IMU: Immune
IS: Immune score
KICH: Kidney chromophobe
KIRC: Kidney renal clear cell carcinoma
KIRP: Kidney renal papillary cell carcinoma
KRT: Keratinization
LGG: Low-grade glioma
LIHC: Liver hepatocellular carcinoma
LoF: Loss-of-function
LUAD: Lung adenocarcinoma
LUSC: Lung squamous cell carcinoma
MESO: Mesothelioma
NCG: Network of cancer genes
NK: Natural killler
OAC: Oesophageal adenocarcinoma
OSCC: Oesophageal squamous cell carcinoma
OV: Ovarian serous cystadenocarcinoma
PAAD: Pancreatic adenocarcinoma
PCPG: Pheochromocytoma and paraganglioma
PKN: Prior knowledge network
PRAD: Prostate adenocarcinoma
READ: Rectum adenocarcinoma
ROC: Receiver operating characteristic
SARC: Sarcoma
SKCM: Skin cutaneous melanoma
SNV: Single nucleotide variant
STAD: Stomach adenocarcinoma
TF: Transcription factor
TGCT: Testicular germ cell carcinoma
THCA: Thyroid carcinoma
TIME: Tumour immune microenvironment
TIS: Tumour inflammation signature
TMB: Tumour mutational burden
TOB: TIME oncogene burden
TTB: TIME tumour suppressor burden
UCEC: Uterine corpus endometrial carcinoma
UCS: Uterine carcinosarcoma
UVM: Uveal melanoma
VAF: Variant allele frequency
