# Supplementary material for: Mechanistic insights into the interactions between cancer drivers and the tumour immune microenvironment
Source: Genome Med. 2023 Jun 5;15:40. doi: 10.1186/s13073-023-01197-0 (PMC10240791; doi:10.1186/s13073-023-01197-0)
Supplement: Supplementary file 1 — Additional file 1: Figure S1. Input preparation for TIME driver identification. Figure S2. Prediction of ICB response in 32 cancer types. Figure S3. CNA profile clustering of HNSC samples. [file 13073_2023_1197_MOESM1_ESM.docx]

**Supplementary Figures**

**Mechanistic insights into the interactions between cancer drivers and the tumour immune microenvironment.**

**Figure S1.** Input preparation for TIME driver identification

**Figure S2.** Prediction of ICB response in 32 cancer types

**Figure S3.** CNA profile clustering of HNSC samples

**Figure S1.** Input preparation for TIME driver identification


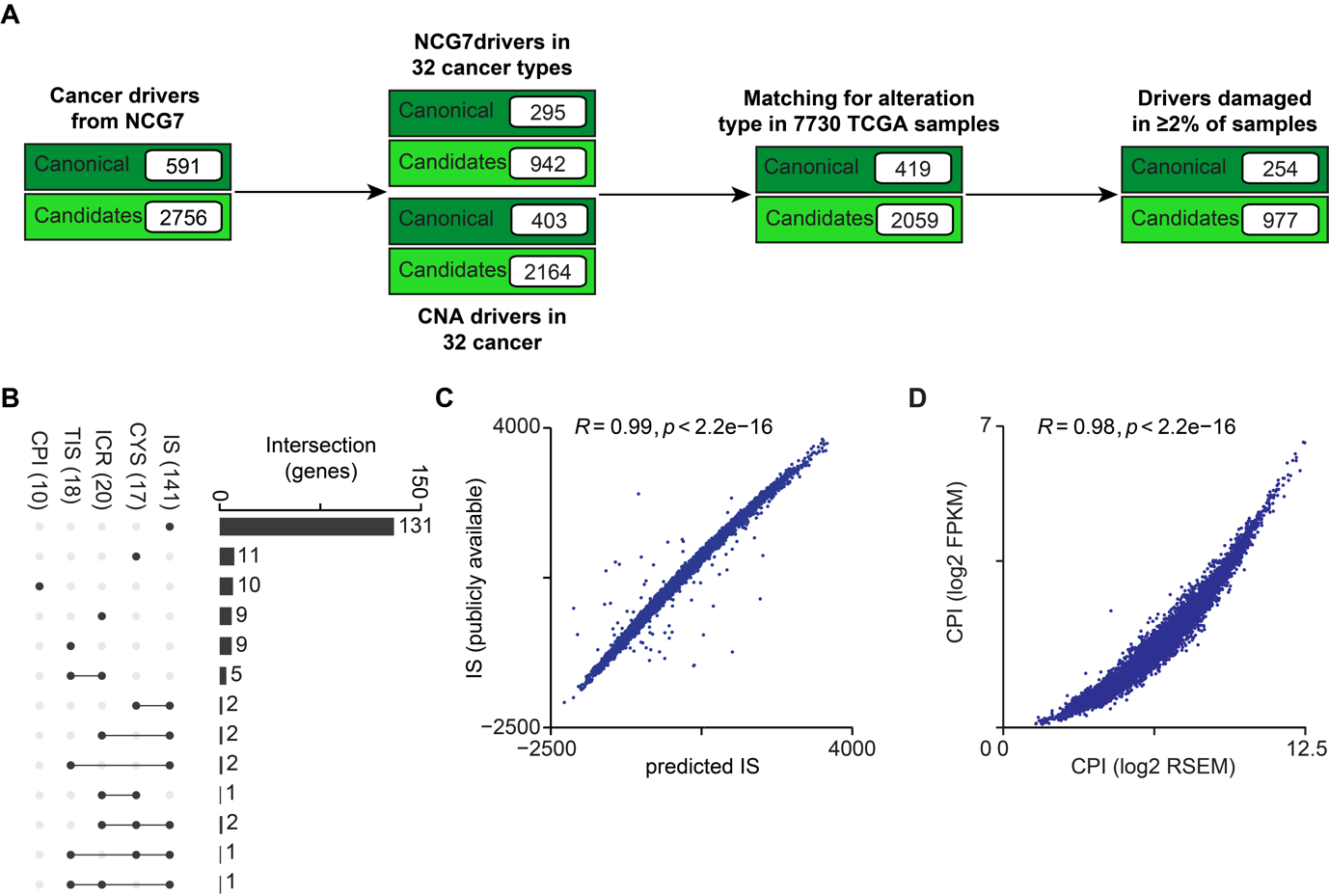


**A.** Approach for deriving cancer-specific drivers. A total of 591 canonical and 2756 candidate drivers were collected in the NCG v.7 database (http://www.network-cancer-genes.org) and mapped to (1) the 32 TCGA cancer types and (2) focal CNA events. Resulting drivers were mapped to damaged genes in 7,730 TCGA samples and only kept only if the alterations matched their mode of action. Finally, drivers damaged in at ≤2% of samples in each cancer type were removed.

**B.** Genes shared across five TIME feature signatures. Number of genes in each signature is shown in brackets.

Correlation between publicly available and predicted IS (**C**) and CPI (**D**) values. Spearman correlation coefficients and associated p-values are shown.

CNA = copy number alteration, TIME = tumour immune microenvironment, IS = immune score, CYS = cytotoxicity score, ICR = immunologic constant of rejection, TIS = tumour inflammation signature, CPI = cancer-promoting inflammation.

**Figure S2.** Prediction of ICB response in 32 cancer types.

**
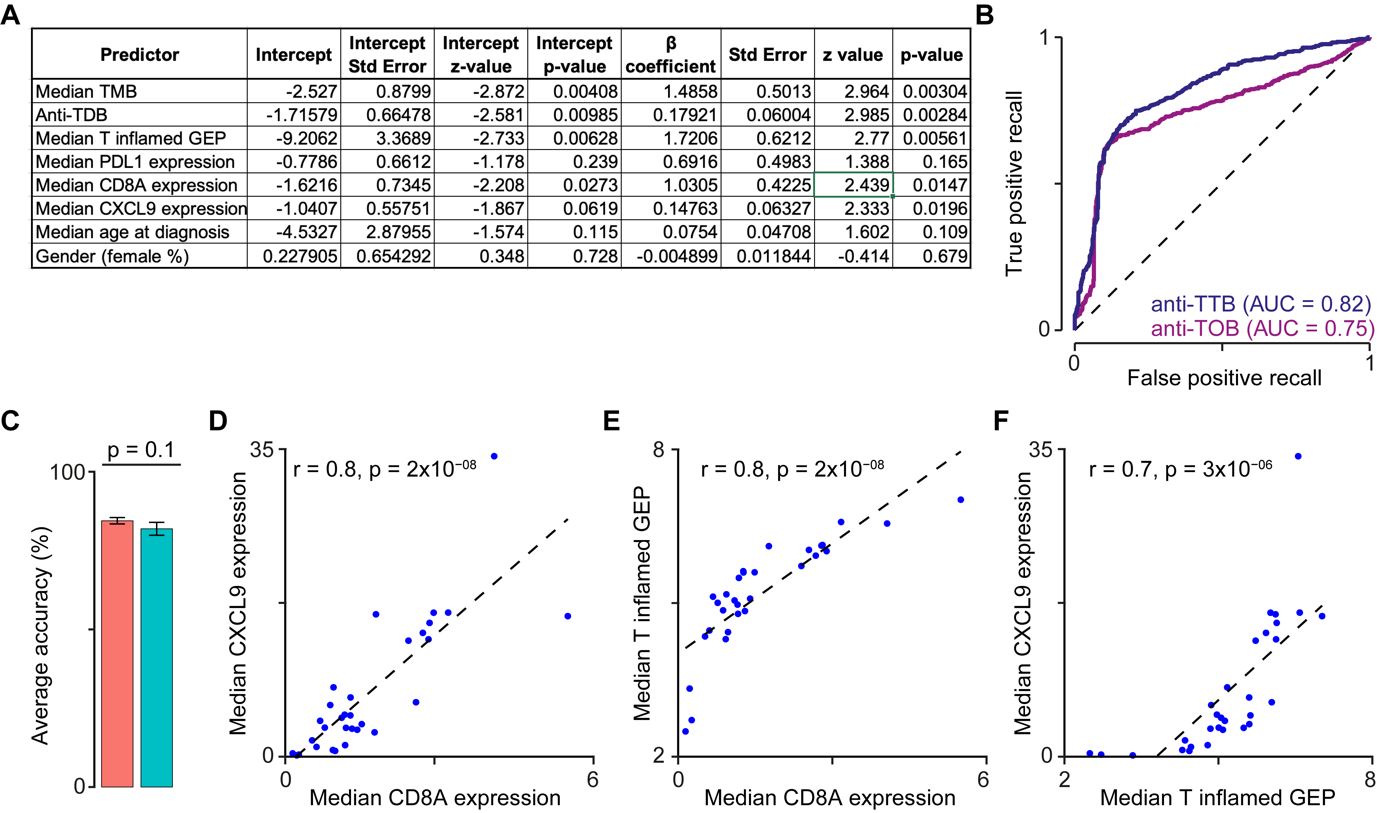
**

**A.** Parameters for Bayesian logistic regression models predicting ICB response across 32 cancer types using eight predictors. Expression -based predictors for *CXCL9*, *PDL1*, *CD8A* genes were calculated as median FPKM gene expression values in 6,921 TCGA samples. T-cell inflamed gene-expression profile (GEP) was measured as described in [1] and the median value was calculated in each cancer type. Clinical data were available for 6,901 samples and were used to derive gender and median age at diagnosis in each cancer type.

**B.** ROC curves comparing the performance of anti-TOB or anti-TTB in predicting response to ICB. Recall rates and AUCs were calculated across 100 cross-validations.

**C.** Average accuracy measured as proportion of true positives with a 95% confidence interval in the training and prediction sets across 100 cross validations using TMB as a predictor of ICB response. Statistical significance was measured using a paired t-test.

Pearson’s correlations between *CD8A* and *CXCL9* expression (**D**); *CD8A* expression and T inflamed GEP (**E**); and T inflamed GEP and *CXCL9* expression (**F**) in 31 cancer types, excluding COAD-MSI.

AUC = area under the curve, anti-TDB = antitumour TIME driver burden, GEP = gene expression profile, ICB = immune checkpoint blockade, TOB = TIME oncogene burden, TTB = TIME tumour suppressor burden.

**Figure S3.** Annotation of the extended HNSC cohort.


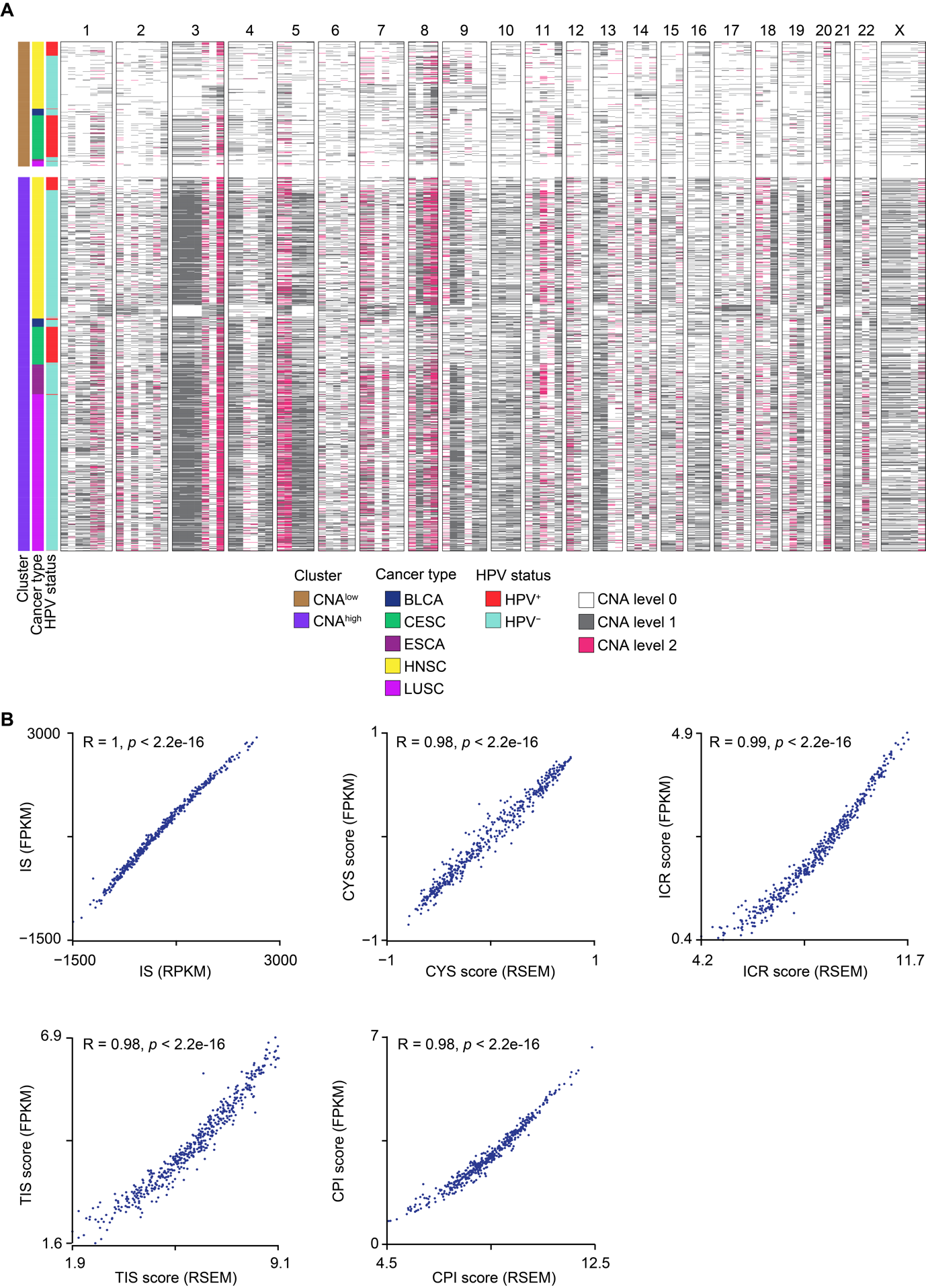


**A.** CNA profile clustering of TCGA squamous cancers and HNSCs from CPTAC. CNA levels indicate the amplitude of CNA as assessed with GISTIC2.

**B.** Correlation between publicly available and predicted IS, CYS, ICR, TIS and CPI values. Spearman correlation coefficients and associated p-values are shown. Spearman correlation coefficients and associated p-values are shown.

CNA= copy number alteration, CPTAC = Clinical Proteomic Tumour Analysis Consortium, Chr = chromosome, HPV = human papillomavirus. TCGA abbreviations are listed in **Additional File 2: Table S1**.

**References**

1. Ayers M, Lunceford J, Nebozhyn M, Murphy E, Loboda A, Kaufman DR, Albright A, Cheng JD, Kang SP, Shankaran V, et al: **IFN-γ-related mRNA profile predicts clinical response to PD-1 blockade.** *J Clin Invest* 2017, **127:**2930-2940.
